# The Tat-Aβ1-6A2V(D) peptide against AD synaptopathy

**DOI:** 10.18632/oncotarget.14604

**Published:** 2017-01-12

**Authors:** Lucia Buccarello, Tiziana Borsello

**Affiliations:** IRCCS -Istituto di Ricerche Farmacologiche “Mario Negri” and Department of Pharmacological and Biomolecular Sciences, University of Milan, Milan, Italy

**Keywords:** synaptic dysfunction, neuroprotection, cell permeable peptide, neurodegenerative diseases, Neuroscience

Synaptopathy is an increasingly popular term used to define a cellular event occurring in an early stage of many neurodegenerative, neurodevelopmental and psychiatric disorders. Such synaptic dysfunction is closely related to cognitive impairment. Nowadays, it is assumed that neurodegenerative diseases, including Alzheimer's disease (AD), are synapse-related pathologies (defined as *“synaptopathy”*) in which misfolded proteins lead to synaptic dysfunction and loss, an event that precedes extensive aggregation of proteins (such as Aβ-oligomers, Phospho-Tau and synuclein) in the brain parenchyma. However, the relationship between protein aggregation and synapses loss remains unclear. Among brain diseases, AD synaptopathy, occurring at a very early stage of the pathology and it is clearly detectable already in patients with mild cognitive impairment (MCI), is the most studied.

The spine-pathology represents a key event of this disease and offer an intriguing possibility to tackle AD. According to the amyloidogenesis hypothesis, altered Aβ species are the primary cause of spine dysfunction and find a way to prevent Aβ toxicity represents an important target for therapeutic intervention against *synaptopathy*.

**Figure 1:**
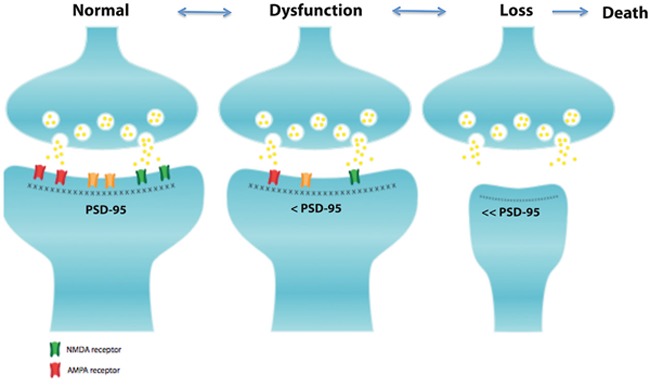
Schematic diagram of the general structure of the excitatory synapses Excitatory synapses are composed by the pre- and post-synaptic elements. In the post-synaptic element there is a specialization of the cell membrane called the postsynaptic density region (PSD), this zone contains a large number of scaffolding proteins. Among these proteins, PSD-95 is the more abundant and plays an important role in the localization of AMPA (red channels) and NMDA (green channel) receptors. Slight perturbations of the spine architecture lead to spine dysfunction and subsequently loss. The mechanism underling synaptic dysfunction is characterized by an initial reversible stage with a slight reduction of PSD-95 protein, NMDA and AMPA receptors that induces a synaptic dysfunction. Afterwards, a major decrease of PSD-95 occurs inducing an atrophy of the post-synaptic element that generate synaptic loss and eventually neuronal death.

Recently, clinical observations have shown that a human mutation in the APP protein (A673V mutation) induces an early onset AD-type dementia in homozygous carriers, while heterozygous carriers are unaffected [1]. In the homozygous patients for A673V mutation, Aβ species were mutated and the APP cleavage generated Aβ mutated oligomers (Aβ_1-42_A2V), while in heterozygous the Aβ oligomers were a mix of both Aβ_1-42_ wildtype and Aβ_1-42_A2V mutated species. To better understand the impact of the A673V mutation in AD, we analyzed the synapto-toxic effect of oligomers formed by the aggregation of different Aβ peptides: 1) the wildtype Aβ_1-42_ wt, 2) the mutated Aβ_1-42_A2V and 3) the combination of the two species: Aβ_1-42_MIX (Aβ_1-42_wt and Aβ_1-42_A2V) in a well characterized in vitro model of synaptopathy [2]. We proved that Aβ_1-42_A2V is more toxic than Aβ_1-42_wt oligomers and induce a more severe synaptic injury in hippocampal neurons. This result is in agreement with the human pathology of the AD A673V-homozygous carriers. Interestingly, the combination of wild type and mutated peptides (Aβ_1-42_MIX) did not exert any synaptic toxicity, confirming that the combination of both species, Aβ_1-42_ wt and Aβ_1-42_A2V peptides, hinders the Aβ-induced-toxicity and counteracts synaptopathy in hippocampal neurons. Importantly, this is in accordance with the fact that the A673V-heterozygous carriers do not develop the disease. This suggests that the Aβ1-6A2V peptide is able to neutralize the toxicity of Aβ_1-42_wt. We hypothesize that the Aβ1-6A2V peptide (composed of the first 6 amino acids with the A673V human mutation sequence) could work as a β-sheet breaker peptide neutralizing the neurotoxic effect of oligomers on dendritic spine. This assumption is also validated by the different chemical-physical characteristics of the Aβ_1-42_A2V/Aβ_1-42_wt MIX. For example, these oligomers have different aggregation kinetic and produce smaller oligomers, which are unstable and not toxic [3]. To test the ability of Aβ1-6A2V peptide to neutralizing Aβ_1-42_wt toxic effect, we synthetized the short peptide Aβ1-6A2V and we tested its ability to counteract the Aβ_1-42_wt toxicity in hippocampal neurons. We found that the Aβ_1-42_A2V peptide exerted a neuroprotective effect against spine injury/loss, as expected. Intrigued by this in vitro neuroprotection, we aimed to validate the Aβ1-6A2V neuroprotective action *in vivo* and we generated a *“bioactive cargo”* by linking the six residues of A673V mutation (Aβ1-6A2V) with the TAT sequence (=Aβ1-6A2VTAT(D)) that allowed cell membrane, as well as Blood Brain Barrier, penetration. Noteworthy, the *in vivo* acute treatment with Aβ1-6A2VTAT(D)) conferred neuroprotection against synaptopathy in AD TgCRND8 mouse model [4], representing an innovative therapeutic tool to prevent AD. In another study, Di Fede and colleagues, performed a chronic treatment with the same peptide, Aβ1-6A2VTAT(D) in a different mouse model, the APP_swe_/PS1ΔE9. They showed that following a short-term treatment (2.5 months), the peptide prevented cognitive deterioration, Aβ aggregation and amyloid deposition in brain, while the longer chronic treatment (5 months) rescued the cognitive impairment, attenuating the effect on Aβ production, but increasing amyloid burden. Such exacerbation of the amyloidogenesis was correlated to the TAT avidity for amyloid boost which may lead to a self-sustained recruitment of Aβ aggregates. This is in agreement with another study where the lentiviral TAT expression-construct injection into APP/PS1Tg mice increased Aβ oligomers as well as as the number and size of Aβ plaques [5]. On the contrary, the administration of a different TAT peptide, the RI-OR2-TAT, in APP_swe_/PS1ΔE9 mice reduced the Aβ oligomer levels in brain parenchyma as well as amyloid-β burden in cerebral cortex and stimulated neurogenesis [6]. In addition, the TAT-BDNF peptide was able to reduce the Aβ level as well as Tau hyperphosphorylation and prevent spine injury in Tg2576 mice express human Swedish mutant APP (APP^swe^) [7]. Finally, the first TAT chronic treatment was done with D-JNKI1 peptide (JNK inhibitor peptide) against AD in TgCRND8 mice (APP_swe/ind_) [8]. This was successful in reducing the amyloidogenic production of Aβ oligomers, powerfully rescued the synaptic dysfunction reverting completely the cognitive impairment, without increased Aβ plaques size and notably, without major side effects [8]. In summary the Aβ1-6A2VTAT(D) peptide is a powerful approach in preventing synaptopathy in preclinical studies. This first *proof of concept* needs further pharmacological investigations in order to define the safety of the cell permeable peptide (CCP). In fact, the CPP-strategy has been considered as a revolutionary breakthrough, but the CPP toxicity is not yet well understood. The safety is now the major issue and is directly related to CPP toxicity and degradation. It is now imperative to concentrate efforts to ensure translation of these finding to human.
